# 环状RNA在肺癌研究中的进展

**DOI:** 10.3779/j.issn.1009-3419.2018.01.07

**Published:** 2018-01-20

**Authors:** 星宇 成, 洪 申

**Affiliations:** 1 510006 广州，华南理工大学医学院 School of Medicine, South China University of Technology, Guangzhou Higher Education Mega Center, Guangzhou 510006, China; 2 510515 广州，南方医科大学基础医学院病理学系 Departments of Pathology, School of Basic Medical Sciences, Southern Medical University, Guangzhou 510515, China

**Keywords:** 环状RNA, 非编码RNA, MicroRNA海绵, 竞争性内源RNA, 肿瘤, Circular RNA, Non-coding RNA, MicroRNA sponge, ceRNA, Cancer

## Abstract

肺癌（lung cancer）的发病率及死亡率在我国双居首位，近年来针对肿瘤驱动基因和免疫检查点的靶点治疗取得了振奋人心的成果。环状RNA（circular RNA, circRNA）是一类具有环形结构的RNA分子，研究发现其与肿瘤的分期、淋巴结转移等关系密切，在生理过程和疾病中具有特殊的生物学功能。其高度稳定性和特异性使之有望成为肿瘤潜在的预测和治疗靶点。目前环状RNA在肺癌中的生物学功能和调控机制仅有少量研究报道。本文对环状RNA的研究历史、生源机制、生物学功能以及其在肿瘤，尤其是肺癌研究中的进展作一综述，以期为环状RNA在肺癌中的研究提供理论依据和新的思路。

## Abstract (en)

Lung cancer is the most commonly diagnosed cancer and the leading cause of cancer death in China. In recent years, therapies for oncogenedrivers and immune checkpoints have proved inspiring. Circular RNA (circRNA), which is a kind of RNA with covalent ring structure relating to stages and metastasis of cancer, has many special biological functions in physiological processes, diseases and so on. Thus, circRNA is expected to be a potential biomarker for cancer prediction and treatment in view of its high conservation and tissue-specific. However, function analysis and regulatory mechanism of circRNA in lung cancer come so far remains unclear and limited literatures are available. In this review, we highlight the research history, formation mechanism, biological function of circRNA and research progress in cancer, especially in lung cancer. We mean to provide theoretical evidences and new ideas for researches on circRNAs in lung cancer.

据全国肿瘤登记中心估计，2015年中国癌症总发病约429万例，总死亡约281万例，其中肺癌是最常见的癌种，在我国的发病率和死亡率双居首位^[1]^。大多数的肺癌患者在确诊时已有转移，五年生存率极低，仅约16%。非小细胞肺癌（non-small cell lung cancer, NSCLC）在肺癌中最为常见，约占总数的80%-85%。传统的手术和放化疗治疗对晚期肺癌都收效甚微，近十余年间，针对肺癌驱动基因和免疫检查点的靶点治疗异军突起。靶向*EGFR*、*ALK*、*KRAS*等驱动基因和PD-1、PD-L1、CTLA-4等免疫检查点的靶点抑制剂，在晚期肺癌的临床试验中效果显著^[2]^。尽管如此，继发性耐药等问题迫使人们不断找寻新的治疗方法。当务之急，深入的机制研究以及新的靶点发现乃是癌症研究的主旋律。随着高通量测序的发展和算法的不断更新，大量非编码RNA被发现在人体中执行多种生物学功能，参与了肿瘤等多种疾病的发生发展过程^[3]^。不同于线性RNA（linear RNA），环状RNA是一类不具有5’和3’末端头尾结构，以共价键形成环状结构的RNA分子。研究发现环状RNA不易被核酸外切酶RNase R降解，半衰期达到48h以上，使得其能稳定存在于真核细胞细胞质中，且具有高度保守性和组织、时序、疾病特异性，从而有望成为潜在的肿瘤诊断标志物和治疗靶点^[4]^。

## 1 环状RNA简介

### 1.1 研究历史

早在19世纪70年代，研究者们通过电子显微镜等手段在病毒中观察到单链、闭合、环状结构的RNA分子^[5]^，1979年，美国洛克菲勒大学的Hsu和Coca-Prados M^[6]^在HeLa细胞细胞质中观察到1%-2%的RNA具有环状结构，首次证实真核细胞中环状RNA的存在。在很长的时间里，由于其发现偶然且检测到的表达水平极低，环状RNA被当作无功能的非特异性的转录垃圾而跻身角落。2012年，Salzman等^[7]^利用RNA-Seq技术在儿童急性淋巴细胞白血病患者骨髓、Hela细胞以及人胚胎干细胞（H9）中检测到大量表达颇丰的环状转录RNA分子，由此证实了在人类胚胎干细胞和恶性组织细胞中均广泛存在环状RNA。随后成千上万的环状RNA被发现，已证实在人类各细胞类型（hg19）、黑腹果蝇（dm3）等生物体中均广泛存在大量环状RNA，引发了新一轮的研究热潮。

### 1.2 生源机制

早期研究者们对于环状RNA的认识源于偶然发现的“Scrambled Exon”^[8]^，提示环状RNA的来源与基因外显子有着密切的联系。后续的研究表明除大部分的环状RNA由外显子构成，内含子也大量参与了形成^[9]^。人们对环状RNA的生源机制尚不明确，研究表明单个基因位点可以通过非经典可变反向剪接（alternative back-splicing junctions）及经典可变剪接（alternative splice junctions）产生多种类型的环状RNA^[10]^。2013年，Jeck等^[4]^提出外显子环化形成环状RNA的两种模型，一种是“套索驱动环化”（lariat-driven circularization），即基因外显子共价结合构成环化；另一种是“内含子配对驱动环化”（intron-pair-driven circularization），即两个内含子互补配对形成环状结构。随后两种模型皆由剪接体剪切剩余内含子形成环状RNA。2014年，Zhang等^[11]^利用全基因组分析和重构circRNA证实了除了ALU重复序列可以介导外显子环化外，内含子的互补序列也能发挥此功能。2015年，Ivanov^[12]^及其团队同样证实了这一机制。另外，研究表明RNA结合蛋白Quaking能够在上皮-间质转化（epithelial-mesenchymal transition, EMT）过程中结合前体mRNA（pre-mRNA）的特定位点，促进环状RNA的形成^[13]^。结合现有研究，根据环状RNA成环方式不同将环状RNA分为以下几类（图 1A）：外显子反向剪接成环（exonic circRNA, ecircRNA）^[10]^、保留内含子的转录本反向剪接（exon-intron circRNA, EIcircRNA）^[14]^、内含子反向互补配对（circular intronic RNA, ciRNA）^[15]^。研究还发现前体tRNA能剪切成环形成tricRNA（tRNA intronic circRNA）^[16]^。此外，研究人员以白血病中的PML/RARα基因为研究对象，在其染色体易位上发现了2个融合环状RNA（fusion circRNAs, f-circRNA），体内试验表明融合环状RNA能促进细胞转化和肿瘤生长^[17]^。总之，环状RNA的生源机制有待进一步探索并加以明确。

**1:**
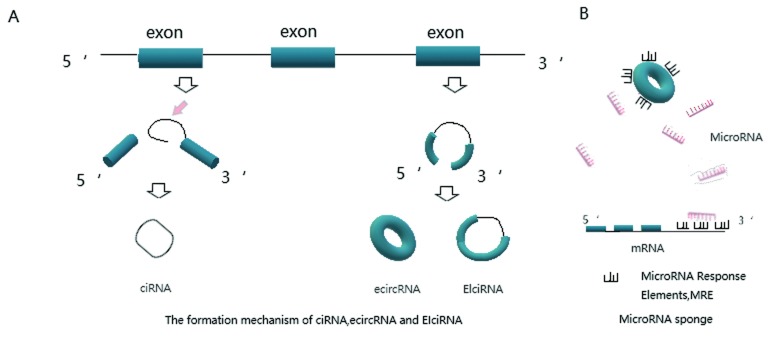
环状RNA的形成机制和MicroRNA海绵功能。A：环状RNA的形成机制示意图：内含子反向互补配对（ciRNA）、外显子反向剪接成环(ecircRNA)、保留内含子的转录本反向剪接(EIcircRNA)；B：MircoRNA结合mRNA能促进其降解，而环状RNA和前体mRNA上均含有MRE反应元件（MicroRNA Response Element，MRE），能够竞争性结合MicroRNA。从而削弱MircoRNA对mRNA的负反馈抑制作用。 Formation mechanism and function of MicroRNA sponge of circular RNA. A:Abridged general view for formation mechanism of circular RNA:circular intronic RNA(ciRNA)、exonic circRNA(ecircRNA)、exon-intron circRNA(EIcircRNA); B: MircoRNA could promote the degradation of mRNA through combination. Meanwhile circRNA contains MRE response elements just the same as mRNA so that competitive binding with MicroRNA is exist. Thus, circRNA could weaken the negative feedback of MicroRNA on mRNA.

### 1.3 生物学功能

#### 1.3.1 MicroRNA海绵

MicroRNA（miRNA）是一类线性的非编码RNA，为基因表达的重要转录后调控因子，能够竞争性结合靶mRNA并促进其降解（图 1B）等，影响生理以至疾病的方方面面。2013年，Hansen等^[18]^在人和小鼠大脑中鉴定出环状RNA分子ciRS-7/CDR1as能够显著抑制miR-7的表达，进一步研究发现ciRS-7上包含了70多个miR-7的特异保守结合位点；同时在小鼠睾丸中发现环状RNA Sry拥有10多个miR-138的结合位点，并能特异调控mir-138的表达，证实了环状RNA能够结合靶MicroRNA调控其表达的功能，推翻了环状RNA作为无功能转录垃圾的观点，拉开了功能研究的序幕。Memczak等^[19]^同样证实了ciRS-7/CDR1as的MicroRNA海绵作用，证明CDR1as是miRNA的天然拮抗剂，它与miRNA的结合力是其他转录产物的10倍。同时，Zheng及其团队^[20]^研究发现circHIPK3有9个miRNA的多个结合位点，表明环状RNA的海绵作用可以同时针对多个miRNA。但目前仅有少数的环状RNA已验证具有miRNA海绵作用。环状RNA作为天然的竞争性内源性RNA（competitive endogenous RNA, ceRNA），有望替代抗核苷酸化学药物，调控miRNA的水平，以期调节上下游基因网络，对人类生理和疾病产生影响^[21]^。

#### 1.3.2 RNA蛋白复合物

在ceRNA的竞争网络中，大量研究证实环状RNA和蛋白分别能与miRNA竞争性结合，但环状RNA与蛋白的结合却少有发现。德国的Albrecht Bindereif教授及其团队^[22]^通过甘油密度梯度离心实验观察沉降系数的分布，初步得到环状RNA与蛋白结合的线索证据。随后鉴定出12种能够同RNA结合蛋白（RNA-binding protein, RBP）IMP3结合的环状RNA分子，证实环状RNA能和蛋白结合形成RNA蛋白复合物。Holdt等^[23]^发现circANRIL能与PES1蛋白结合，介导动脉粥样硬化。随后的研究也证实了一些环状RNA可以结合蛋白产生生物学效应。

#### 1.3.3 转录翻译

一直以来环状RNA被当作非编码RNA的一类，既往的研究并没有发现环状RNA在真核细胞内存在翻译活动。而最新的研究表现，环状RNA中含有丰富的m6A甲基化修饰。YTHDF3是m6A的识别蛋白，能够与环状RNA的修饰位点结合，并募集eIF4G2和其他翻译起始因子，从而启动环状RNA的翻译^[24]^。Pamudurti等^[25]^在果蝇的大脑组织中通过核糖体印记分析鉴定出circMBL的终止密码子处有核糖体结合，并通过蛋白质谱分析得到了环状RNA翻译蛋白的直接证据。这使得环状RNA或许有可能成为新的一类信使RNA分子。

#### 1.3.4 其他功能

环状RNA还能调节基因转录^[14]^，作为pre-mRNA的剪接调节器影响蛋白质的合成^[26]^。然而其生物学功能研究仍处于起步阶段，需要更深入的研究加以阐释。

## 2 环状RNA与肿瘤

环状RNA广泛参与了生理和疾病的发生发展过程，在心血管系统、消化道系统、血液循环系统和神经系统等疾病中均发挥着重要作用，在胚胎、外泌体中也有丰富的表达，在肿瘤方面亦如此。研究发现环状RNA大多以MicroRNA海绵的身份在肿瘤的发生发展中发挥作用^[27]^（图 2^[28-59]^）。其中CDR1as（ciRS-7）、circ-HIPK3等环状RNA在多种类型的肿瘤组织以及非肿瘤性疾病中均有丰富表达，相应的功能机制研究受到了广泛的关注。

**2:**
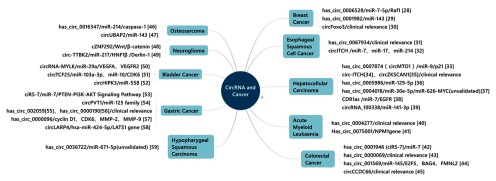
环状RNA与肿瘤。部分环状RNA已经验证在肿瘤中发挥生物学功能，其中大多数的环状RNA在肿瘤的发生发展中发挥着MicroRNA海绵的作用。 CircRNA and cancer. Some circRNAs have been validated with biological functions in cancer and most of them play the role of MicroRNA sponge in kinds of cancer.

### 2.1 CDR1as

小脑变性相关蛋白1反义转录产物CDR1as是最早发现具有MicroRNA海绵功能的环状RNA。CDR1as结合miR-7的经典组合已证实在神经退行性疾病、糖尿病、动脉粥样硬化中发挥重要调控作用^[60]^。研究表明CDR1as/miR-7在结直肠癌、肝癌等肿瘤中调控其生物学行为。既往研究已揭示miR-7可以直接作用于肿瘤相关信号通路中的重要蛋白，如脑胶质瘤中miR-7/EGFR和PKB/IRS-1和IRS-2通路，乳腺癌中miR-7/Pak1和HOXD10通路和在肝癌中的miR-7/PI3K和Akt通路等，提示CDR1as可通过miR-7海绵间接调控miR-7相关通道^[61]^，影响肿瘤的生物学行为。

### 2.2 circHIPK3环状RNA分子

circHIPK3在多种类型的肿瘤和正常组织中差异表达明显。研究人员发现circHIPK3可以结合多种MicroRNA发挥生物学效应：circHIPK3/ miR-124在人体细胞中调控其生物学活动；circHIPK3/miR-30a/表皮生长因子EGFC、FZD4和WNT2在糖尿病视网膜血管功能障碍中具有重要作用^[62]^；circHIPK3/miR-558/HPSE在膀胱癌中调控其迁移、侵袭和血管生成^[52]^。

## 3 环状RNA与肺癌

前人通过高通量测序已经在肺癌组织和细胞系中发现了成千上万的环状RNA，但其在肺癌中的具体功能和作用机制却少有研究。总体而言，已有的环状RNA在肺癌中的研究大致可分为生物标志物研究和功能机制研究。

### 3.1 生物标志物

Yao等^[63]^发现在NSCLC中circRNA-100876高表达与肺癌的淋巴结转移和肿瘤分期的关系密切，且circRNA 100876高表达的NSCLC患者的总体生存时间明显缩短。Zhu等^[64]^利用基因芯片在肺腺癌中筛选出具有差异表达的环状RNA，其中hsa_circ_0013958在组织细胞、血浆中均显著上调，且与淋巴结转移和肿瘤分期具有统计学意义。Zhao等^[65]^对4例非吸烟型早期肺腺癌的肿瘤组织和癌旁组织进行高通量测序，找到300多个在肿瘤组织中差异表达的环状RNA，并通过RT-qPCR验证了其中的5个，结果与芯片结果相符，为早期肺腺癌的诊断和治疗研究提供了潜在的靶点。Luo等^[66]^研究注意到hsa_circ_0000064在肺癌组织及肺癌细胞系A549和H1229中表达上调，其异常表达与肿瘤淋巴结转移和TNM分期等临床特征密切相关。

### 3.2 功能机制

Jin等^[67]^对肺癌病例和健康对照组的血液样本进行RNA测序，检测了miRNA、circRNA和mRNA以及长非编码rna（lncRNA），通过通路富集分析，建立了以MiRNA为目标的内源性竞争网络，筛选重要节点进行生物信息学预测，构建了转录因子调控网络。Hansen等^[68]^研究发现CDR1as能通过结合miR-7发挥对肿瘤的抑制作用，在肺癌中亦如此。Wan等^[69]^对78例肺癌患者的癌组织和癌旁组织进行了circ-ITCH的检测，结果显示circ-ITCH在肺癌中表达明显减少。在细胞实验中，circ-ITCH异常表达可以显著影响其亲代癌抑制基因ITCH的表达，抑制肺癌细胞增殖。进一步实验表明circ-ITCH可充当miR-7和mir-214海绵，增强ITCH基因的表达，从而抑制Wnt/β-Catenin信号通路的激活。田芳^[70]^及其团队在NSCLC细胞系H1299、H827、H1975、H2170、H520、H1650中均检测到circ-HIPK3的表达，且NCI-H2170表达量最高，NCI-H1299表达量最低。实验表明在NCI-H1299和NCI-H2170中，circHIPK3能结合miR-379调控类胰岛素样生长因子IGF1的表达水平，促进细胞增殖。利用肉桂醛干预抑制Wnt/β-Catenin信号通路，发现hsa_circ_0043256显著下调，并能够作为miR-1252海绵削弱肉桂醛的抑制作用。进一步研究发现ITCH基因表达与hsa_circ_0043256成正相关，提出了肉桂醛干预NSCLC hsa_circ_0043256/ miR-1252/ITCH轴的作用新机制^[71]^。Zhu等^[64]^研究表明hsa_circ_0013958能与miR-134结合调控细胞周期蛋白D1（cyclin D1）的表达。

### 3.3 启示

迄今为止，越来越多的实验证据证实环状RNA具有稳定特性和组织、时序、疾病特异性，这使得环状RNA相较于miRNA似乎更具有生物标志物和治疗靶点价值。从已有的关于肺癌中的环状RNA来看，首先，由于高通量测序成本代价高，已有研究用于测序的样本量较少，而后续试验验证所用总体样本量虽然基本可以达到统计学要求，但是具体到肺癌各组织分型的标本数据明显欠缺，使得横向比对各组织分型中的环状RNA的数据明显缺失或者不可靠；肺癌发生发展时间轴（正常-癌前病变-早期-晚期肺癌）纵向比对环状RNA表达差异和机制研究由于诊断和取材等限制难以实现。期待随着高通量技术的不断成熟、成本的降低以及算法的不断完善，大范围大样本的肺癌环状RNA研究能成为现实。其次，研究发现环状RNA在人体血浆、外泌体等也广泛存在，而现有的肺癌环状RNA研究利用的标本种类过于单一，对于肺癌循环血浆及外泌体等的环状RNA研究少有涉及，不同标本类型间的差异研究更属罕见。再者，环状RNA分子具有多种重要的生物学功能，且目前为止仅有少量的环状RNA经验证具有MicroRNA海绵功能。此外现有肺癌环状RNA研究几乎都致力于研究一个新的环状RNA分子的MicroRNA海绵功能，使得有关研究雷同而不够全面、不够深入。ceRNA内源性竞争机制的复杂调控网络亟需纳入更多的因子（如mRNA、蛋白等）来丰富和完善。此外，集中对CDR1as、circHIPK3等明星环状RNA分子的更深层次机制和功能的挖掘有助于我们更深刻地了解环状RNA的特性。最后，环状RNA性质稳定，半衰期较长且不易降解的特性赋予了针对环状RNA进行RNA药物研发的可行性；环状RNA内源性竞争机制为替代抗核苷酸化学治疗的研究提供了理论基础。目前环状RNA的研究尚处于起步阶段，功能和机制研究尚不明确，基于环状RNA的RNA药物研发任重而道远。

## 4 展望

靶点治疗已成为癌症治疗研究的新宠。肺癌作为我国发病率和死亡率双居首位的癌种，尽管EGFR、PD-L1等靶点治疗在临床试验中大放光彩，但是我们也必须看清局限的治疗目标和缓解率以及继发性耐药等问题迫使我们不能忽视对肺癌的基础研究，还需寻找新的预测、诊断和治疗靶点。环状RNA在真核细胞细胞质中广泛存在，相对于线性的RNA具有高度稳定特性和组织、时序、疾病特异性，在肿瘤中发挥着一系列生物学功能，使其具备作为肿瘤预测、诊断和治疗靶点的潜能。但环状RNA的研究尚处于起步阶段，其机制及生物学功能尚不明确，在肺癌中的研究更是屈指可数，有待更多更深层次的研究来阐明。在肺癌的环状RNA研究中，我们还需横向扩大各组织病理分型的样本数量和样本类型以比较不同肺癌分型和样本类型的环状RNA差异，或者纵向延伸肺癌发生发展时间轴以比较肺癌发生发展中的环状RNA差异。实现这些目标，需要强大的人力、物力、财力的支撑，相信随着高通量测序技术的完善和成本的降低，以及算法的不断完善，势必会准确预测出更多的环状RNA分子，实现大规模大样本的环状RNA研究，为探索肺癌的生物学标志物以及后续的功能研究打下基础；环状RNA在肺癌中的功能机制研究也不会仅仅局限于MiRNA海绵，而是在ceRNA内源性竞争机制的海洋里大放异彩，奠定内源性竞争机制替代抗核苷酸化学治疗的基础。RNA药物治疗肿瘤从基础走向临床或将成为必然。总而言之，环状RNA是一类新兴的有望成为肿瘤潜在的预测、诊断和治疗靶点的RNA分子，具有极高的研究潜能和价值。
